# Fastq-dupaway: a fast and memory-efficient tool for deduplication of single- and paired-end NGS data

**DOI:** 10.1038/s41598-025-28948-w

**Published:** 2025-11-25

**Authors:** A. I. Sigorskikh, M. A. Kompaniets, I. S. Ilnitskiy, G. K. Ryabykh, A. A. Mironov

**Affiliations:** 1 Faculty of Bioengineering and Bioinformatics, Lomonosov Moscow State University, Moscow, 119234 Russia; 2 Vavilov Institute of General Genetics, Russian Academy of Sciences, Moscow, 119991 Russia

**Keywords:** PCR duplicates removal, NGS data processing, Bioinformatics’ tool, Computational efficiency, Biological techniques, Computational biology and bioinformatics

## Abstract

The rapid emergence of large-scale next-generation sequencing (NGS) data has created a growing demand for efficient preprocessing tools. Removal of polymerase chain reaction (PCR) duplicates is a critical step in many NGS data processing pipelines to reduce amplification bias. Currently, numerous *de novo*-based PCR duplicate removal tools are available, which cluster identical or highly similar reads without reference genome alignment. Practical application of such programs to large-scale NGS data (100 GB and more) is hampered by significant computational requirements, particularly high computer short-term memory usage comparable to the original file sizes. Processing large datasets generated by modern high-throughput techniques such as Hi-C or RNA-chromatin interaction sequencing can require hundreds of gigabytes of RAM, posing an exceptionally high computational demand. Here, we present Fastq-dupaway as a new tool for efficient PCR duplicate removal from both single-end and paired-end sequencing data (https://github.com/AndrewSigorskih/fastq-dupaway). Its key innovation lies in its primary operational modes, which are designed to use a small, parameterizable amount of RAM (2–10 GB) independent of input data size, at the cost of requiring approximately 2$\times$ the input file size in disk space. This enables the processing of very large datasets even on standard personal computers. Fastq-dupaway matches or exceeds (by up to threefold) the processing speed of major *de novo* deduplication tools, while maintaining the same level of duplicate removal.

## Introduction

Currently, next-generation sequencing (NGS) methods are widely used in various biological applications^1^. The sequencing process involves several key steps: sample preparation, nucleic acid fragmentation, amplification, and sequencing. Each stage introduces systematic errors that can, to varying degrees, impact the quality of the data and its interpretation^2^. In particular, during amplification (also known as polymerase chain reaction, PCR), copies of the original DNA molecules (PCR duplicates) are generated, which can constitute a substantial fraction of the sequencing data (in some cases, tens of percent of the total)^3^. The extent to which PCR duplicates distort sequencing data remains a subject of ongoing debate^3–6^.The core of this debate centers on whether deduplication is an essential step to remove technical artifacts that bias quantitative analyses, or whether it can inadvertently remove biologically meaningful signal in certain experimental contexts, such as those with inherently low diversity.

In addition to solutions at the sample preparation level, such as use of unique molecular identifiers (UMIs) to tag template molecules prior to amplification, a wide range of bioinformatics tools have been developed to identify and remove PCR duplicates from sequencing data. The computational analysis of UMI-based data is performed by specialized tools, such as those described in^7^ and BBTools Clumpify^8^, and their analysis falls beyond the scope of this work. All deduplication tools can be classified into two categories: “alignment-based” and “*de novo*-based”^9^. The key component of alignment-based methods is the alignment of reads to a reference genome or transcriptome, which makes these methods dependent on the availability and quality of the reference genome/transcriptome. Among the most well-known tools in this category are Picard MarkDuplicates^10^ and SAMtools markdup^11^. In contrast, *de novo*-based deduplication methods identify and cluster identical or highly similar reads without aligning them to a reference genome. Numerous tools implement this approach, including FastUniq^12^, Seqkit rmdup^13^, BBTools Clumpify^8^, CD-HIT-DUP^14^, Fastx Toolkit Collapser^15^, and others^16–24^.

Read mapping to a reference genome is traditionally the most time-consuming step in NGS data preprocessing, which prompts scientists to apply approaches to early removal of PCR duplicates to optimize computational processes. However, transitioning to *de novo*-based deduplication methods introduces a new challenge: these methods typically require loading the entire dataset into random access memory (RAM), which is feasible for smaller datasets like RNA-seq but becomes a critical limitation for modern datasets containing hundreds of millions of reads. The RAM requirement can escalate to tens or even hundreds of gigabytes (GB) (see section “Comparison with *de novo*-based PCR deduplication tools”), increasing computational costs and rendering processing inaccessible to researchers without access to high-performance computing (HPC) infrastructure. Additionally, some tools alter read identifiers (Fastx Toolkit Collapser) or do not properly handle paired-end reads (FastUniq). Our *de novo*-based deduplication tool, Fastq-dupaway, enables a streaming approach to deduplication with minimal RAM usage, making it feasible to analyze data even on a personal laptop while maintaining accuracy and speed. Fastq-dupaway provides several key features that are absent in many existing *de novo*-based methods, including: (i) support for both single-end and paired-end NGS data; (ii) direct processing of compressed files (e.g., gzip format); and (iii) four distinct modes for PCR duplicate removal.

We compared PCR deduplication tools across various experimental datasets, including Hi-C, ChIP-seq, Whole Genome Sequencing, Exome-seq, RADICL-seq, GRID-seq, ChIRP-seq, and CHART-seq. We demonstrated that existing deduplication tools load entire datasets into RAM, leading to high RAM demands. Additionally, several tools relying on JVM exhibit unstable performance. In contrast, our method enables efficient processing of very large datasets with minimal resource requirements.It is noteworthy that utilizing substantial RAM capacity incurs a significantly higher cost compared to employing disk storage, i.e. upgrading the system with more disk capacity is orders of magnitude cheaper than adding equivalent amounts of RAM, and RAM expansion is restricted by motherboard and CPU limitations.

## Program design and implementation

Fastq-dupaway provides multiple options for flexible workflow customization. The program operates on single-end or paired-end inputs, supports FASTQ and FASTA file formats, and works with both plain and gz-compressed files. The user can choose between two deduplication algorithms: (i) a “sequence-based” mode, which provides control over the upper memory (RAM) usage limit and sequence comparison logic, at the cost of requiring approximately 2$\times$ the input file size in disk space; and (ii) a “fast” mode, which is designed for speed and removes only exact duplicates but does not allow setting a memory usage limit (Fig. S1).

### Sequence-based mode

This mode operates by comparing sequences directly. During execution, memory usage is controlled to remain at or below the user-defined threshold.

First, reads in the input file(s) are sorted by their sequence. The program uses a variation of an “external sort” algorithm similar to merge sort, with time complexity of $O(N\cdot \log N$). In this mode, the program creates temporary files totaling approximately 2x the input file size. The imposed RAM limit does not affect the total disk space usage but directly influences the runtime performance. During the second step, the program reads the sorted input and removes duplicate records in a single pass. The amount of data read and written during I/O operations when running the sequence-based algorithm is 3x the volume of the input data. The logic for defining a “duplicate” supports three options:

- “tight” mode (enabled by default). Only complete sequence duplicates are removed. Sequences of different lengths are automatically considered non-duplicates.
- “loose” mode. This mode produces results identical to those of FastUniq^12^, with both tools being sensitive to the order of input files. However, Fastq-dupaway “loose” achieves this through a more RAM-efficient streaming implementation. During sequence comparison, sequences of different lengths are considered duplicates if the shorter sequence exactly matches the prefix of the longer sequence.
- “tail-hamming” mode. This is an experimental mode based on the proposition that mismatches caused by sequencing errors are more likely to be observed at read ends rather than at the beginnings^25^. In this mode, two reads are considered duplicates if they are adjacent in the sorted file and the Hamming distance between them is no greater than the user-defined threshold.

In the case of paired-end reads, the duplicate detection logic is the same as above with one addition: both the “left” and “right” reads of pair B must be duplicates of the corresponding reads of pair A for pair B to be removed.

#### Fast mode

This mode operates by comparing sequence hashes. During execution, sequences are packed into arrays of 64-bit integers. Memory usage cannot be limited in this mode, and it detects and removes only exact duplicates. However, it is faster than the sequence-based mode and is not disk-intensive.

#### Mode selection guidelines

We recommend using the “fast” mode for relatively small datasets, when rapid results are required, or when operating on a system under high I/O load. Sequence-based modes are more suitable for large datasets on systems with limited computational resources, provided that I/O performance is not a limiting factor. The “tight” mode offers a balanced baseline for deduplication, the “loose” mode represents an efficient implementation of the FastUniq algorithm, and the “tail-hamming” mode can be viewed as a slightly more aggressive variant of the tight mode.

### Comparison with *de novo*-based PCR deduplication tools

Preliminary testing of *de novo*-based deduplication methods revealed that several programs have notable limitations (Table 1). For instance, Fastx Toolkit Collapser accepts input in FASTQ format but outputs a FASTA file with modified read IDs. Seqkit rmdup is limited to single-end reads, while FastUniq processes only paired-end reads, and its output depends on the order of the input files. In contrast, Fastq-dupaway demonstrates flexibility and versatility, effectively handling all these scenarios. Note that both FastUniq and the Fastq-dupaway “loose” mode produce results that are highly dependent on the order of the input files. This behavior may be attributed to the inherent ambiguity in defining duplicate identity when implementing the FastUniq logic, which relies on sequence prefix comparison. For a detailed description of the comparison algorithm with examples, please refer to the manual on GitHub. Different tools possess certain additional features not presented in Table 1. In particular, BBTools Clumpify can identify optical duplicates and perform error-correction.

**Table 1:** Comparison of the characteristics of *de novo*-based PCR deduplication tools.

PCR deduplication tool	Input data (paired-end/single-end/any)	Do read IDs change?	Result is affected by input files order	There is a parameter “N-mismatch”	Are there any customizable options?	Can work with gz-compressed files	Handles ambiguous nucleotides (N)	Primary programming language
Fastq-dupaway “tight”	Any	No	No	No	Yes	Yes	No	C++
Fastq-dupaway “loose”	Any	No	Yes	No	Yes	Yes	No	C++
Fastq-dupaway “tail-hamming”	Any	No	No	Yes	Yes	Yes	No	C++
Fastq-dupaway “fast”	Any	No	No	No	Yes	Yes	No	C++
FastUniq	Paired-end	No	Yes	No	No	No	No	C
BBTools Clumpify	Any	No	No	Yes	Yes	Yes	Yes	Java
CD-HIT-DUP	Any	No	No	Yes	Yes	No	No	C++
Fastx Toolkit Collapser	Single-end	Yes (output in FASTA format)	–	No	No	No	No	C/C++
Seqkit rmdup	Single-end	No	–	No	Yes	No	No	GO

We compared Fastq-dupaway (in its various modes) against five widely used *de novo*-based deduplication tools – FastUniq, BBTools Clumpify, CD-HIT-DUP, Fastx Toolkit Collapser, and Seqkit rmdup – on 15 datasets of varying types and sizes (Table 2). To ensure equitable comparison, all tools were executed on a single CPU core (supporting one thread). CPU time, elapsed time, maximum Resident Set Size (RSS) used by each tool, and the percentage of PCR duplicates removed for each dataset are presented in S1-S8 Tables.

**Table 2:** Characteristics of the NGS datasets used for benchmarking PCR duplicate removal tools. The data are available from https://www.ncbi.nlm.nih.gov/sra.

Protocol type	Dataset	Library layout	Read count	Dataset size (GB)	Organism
RADICL-seq	SRR9201799, SRR9201800	paired-end	141,369,583	36	*Mus musculus*
GRID-seq	SRR3633290, SRR3633291	paired-end	149,560,162	42	*Mus musculus*
ChIRP-seq	SRR1425229	single-end	297,394,120	53	*Homo sapiens*
CHART-seq	SRR10044362	single-end	60,857,691	10.8	*Homo sapiens*
Whole Genome Sequencing	SRR2014554	paired-end	24,627,585	12.5	*Escherichia coli RR1*
Whole Genome Sequencing	SRR19505554	paired-end	175,735,042	104	*Homo sapiens*
Whole Genome Sequencing	SRR19505555	paired-end	201,266,001	112.4	*Homo sapiens*
ChIP-seq (H3K27me3)	SRR8902551	paired-end	31,868,491	12.3	*Mus musculus*
ChIP-seq (H3K4me1)	SRR504934	single-end	31,269,559	5.3	*Homo sapiens*
ChIP-seq (CTCF)	SRR10950502	paired-end	51,668,100	16.5	*Mus musculus*
Hi-C	SRR9675763	paired-end	305,604,544	106.6	*Homo sapiens*
Hi-C	SRR8902547	paired-end	150,153,852	47.6	*Mus musculus*
Hi-C	SRR1658643	paired-end	1,094,811,672	538	*Homo sapiens*
Exome-seq	SRR24907572	paired-end	91,397,861	62.4	*Homo sapiens*
Exome-seq	SRR13232316	paired-end	127,197,855	91.4	*Homo sapiens*

**Fig. 1:**
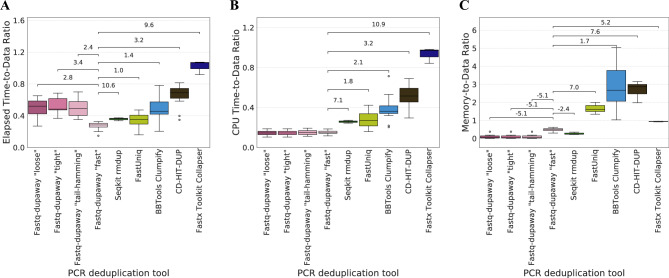
Triple-metric performance comparison of deduplication tools. (**A**) Elapsed Time-to-Data Ratio, (**B**) CPU Time-to-Data Ratio and (**C**) Memory-to-Data Ratio. All ratios were calculated by dividing the absolute metric by the corresponding dataset size (GB). Elapsed and CPU time ratios have units of min/GB; memory ratio is dimensionless. Lower values indicate better performance across all panels. Statistical analysis compared each tool against Fastq-dupaway “fast” mode (8 comparisons per metric) using Welch’s t-test with Benjamini-Hochberg false discovery rate correction. Horizontal connectors highlight statistically significant differences (FDR < 0.05), with covariance-adjusted strictly standardized mean difference (SSMD) values annotated above each connector. A more rigorous statistical evaluation using alternative methods has been provided in Supplementary Note 1.

Fastq-dupaway shows distinct performance characteristics across different metrics. For elapsed time, the “fast” mode was the fastest among all tools tested, whereas the “tight”, “loose”, and “tail-hamming” modes were approximately 1.5 times slower than Seqkit rmdup and FastUniq (Fig. 1A, Fig. S2A, Table S2), reflecting I/O overhead from disk-based operations. In contrast, all Fastq-dupaway modes demonstrate the best performance in CPU time (Fig. 1B, Fig. S2B, Table S1). Memory usage patterns further highlight the tool’s utility. The “tight”, “loose”, and “tail-hamming” modes maintain a consistent low memory footprint of approximately 2 GB, making them suitable for processing large datasets on standard personal computers. The “fast” mode uses less memory than most tools, being outperformed only by Seqkit rmdup, which is limited to single-end reads (Fig. 1C, Fig. S2C, Table S3). Programs like Clumpify and CD-HIT-DUP demand substantial RAM, making them primarily suitable for computational clusters with large RAM capacities (on average, 2–3 times the size of the processed data) (Fig. 1C, Fig. S2C). This limitation became especially apparent during the processing of a large Hi-C dataset (SRR1658643, 538 GB, 1,094,811,672 paired-end reads), where CD-HIT-DUP and FastUniq required approximately 1 TB of RAM. BBtools Clumpify crashed on this dataset when limited to one CPU core, but is stable with four cores. In the same test, Fastq-dupaway in “fast” mode uses 6-fold less RAM than FastUniq and CD-HIT-DUP (Fig. 1C), confirming its advantages when processing large datasets on resource-constrained hardware.

As shown in Fig. 1 (CPU Time-to-Data Ratio), the “fast” mode of Fastq-dupaway demonstrates slightly slower performance compared to the “tight”, “loose”, and “tail-hamming” modes. This relationship, however, is inverted in Fig. 1 (Elapsed Time-to-Data Ratio), where the “fast” mode proves to be the fastest among all. This apparent discrepancy is explained by the fundamental difference in how these modes utilize system resources. The “tight”, “loose”, and “tail-hamming” modes are disk-intensive, employing an external sorting algorithm that generates significant input/output (I/O) operations. While this approach minimizes RAM consumption, the associated disk I/O latency is captured in the elapsed time measurement but not in the pure CPU time. Conversely, the “fast” mode operates primarily in RAM, trading higher memory usage for vastly superior I/O performance. Consequently, its total execution time (elapsed time) is lower, as it avoids the bottleneck of disk access, despite requiring more CPU cycles for its in-memory hashing and deduplication processes.

It is worth noting that while most tools, including all modes of Fastq-dupaway, operate in single-threaded mode, BBTools Clumpify offers multi-threading capabilities. Our comparison of its single-threaded versus multi-threaded execution (Table S14) revealed that multi-threading can provide Clumpify with up to a threefold increase in processing speed.

For the main benchmarking, all programs were run five times on each dataset. As the size of processed data increases, the variability in runtime grows, with this effect being more pronounced for elapsed time compared to CPU time (Fig. S3, Fig. S4, Table S12, Table S13). This discrepancy occurs because elapsed time includes not only computational time (CPU time) but also overhead, such as input/output operations, memory release delays, and other system latencies. Variability in “seq-based” fastq-dupaway modes may depend on the server’s I/O load. In contrast, the variability of other tools, being less I/O-bound, may be attributed to different factors, the detailed assessment and minimization of which fall beyond the scope of typical scientific applications. BBTools Clumpify exhibited the greatest performance instability, especially in RAM utilization (Fig. S5).

**Fig. 2:**
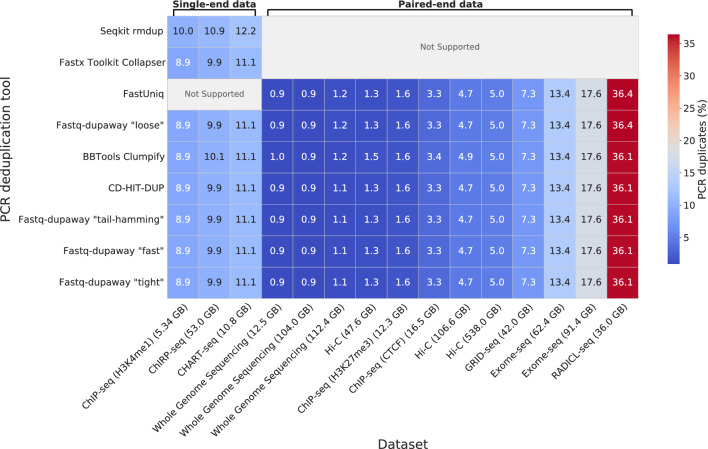
Percentage of PCR duplicates identified by each tool per dataset. PCR duplicates were identified with zero mismatches allowed. For the “Hi-C (538.0 GB)” dataset, BBTools Clumpify was executed in multi-threaded mode. Not Supported: a tool does not support the corresponding data type.

**Fig. 3:**
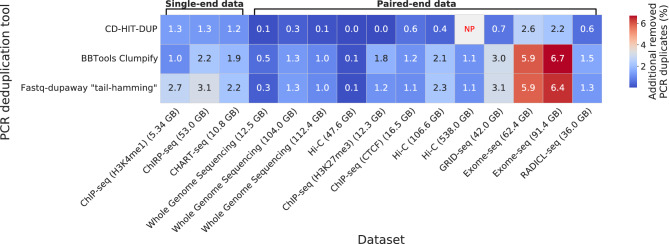
Percentage of additional PCR duplicates removed by each tool per dataset when allowing two mismatches. For the “Hi-C (538.0 GB)” dataset, BBTools Clumpify was executed in multi-threaded mode. NP: not processed due to error.

**Fig. 4:**
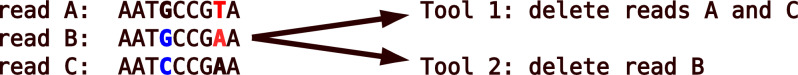
Transitivity problem in PCR duplicate identification with mismatches.

When no mismatches between reads are allowed, different methods show similar efficiency in removing PCR duplicates (Fig. 2, Table S4). However, when accounting for potential differences of a few nucleotides (e.g., two), substantial variability in results is observed across programs, with the proportion of additionally removed reads varying by up to threefold (Fig. 3, Table S5). This variability stems from the fundamental issue of transitivity in identifying PCR duplicates with allowable mismatches and the lack of a single standardized approach to their identification. For example, when using Hamming distance, reads “A” and “B” may differ by one nucleotide, as may reads “B” and “C”, while reads “A” and “C” differ by two. Depending on the algorithm implemented in a particular program, either read “B” or both reads “A” and “C” may be removed (Fig. 4). This inherent transitivity means that when mismatches are allowed, the problem becomes degenerate, and no single ground truth solution exists. Consequently, it is impossible to define or estimate standard Type I and Type II error rates in this context. The final set of removed reads is entirely determined by the specific algorithm employed by each tool. Thus, both “Tool 1” and “Tool 2” in Fig. 4 provide logically valid, yet different, solutions to the same ill-posed problem.

Performance analysis with an allowable mismatch level of two nucleotides shows that Fastq-dupaway in “tail-hamming” mode achieves higher data processing speeds compared to BBTools Clumpify and CD-HIT-DUP, while consistently using exactly 2 GB of RAM, regardless of the data size (Table S6, Table S7, Table S8). At the same time, when processing a large Hi-C dataset (SRR1658643, 538 GB), both BBTools Clumpify and CD-HIT-DUP failed, seemingly due to memory limitations. This result underscores the robustness of the Fastq-dupaway algorithm for processing large datasets while maintaining predictable memory consumption.

### Comparison with the alignment-based PCR deduplication tool

A direct comparison of the accuracy between alignment-based and *de novo*-based PCR duplicate removal methods is inherently challenging, as these approaches rely on fundamentally different principles for duplicate identification. Alignment-based methods utilize genomic coordinates to distinguish true PCR duplicates from distinct molecules that share identical sequences but originate from different genomic loci, particularly in repetitive regions. In contrast, *de novo* methods, which rely solely on sequence identity, cannot make this distinction and may consequently misclassify non-duplicate reads from repetitive regions as duplicates. However, this theoretical advantage of alignment-based methods is often mitigated in practice. In standard short-read analyses (such as RNA-seq or ChIP-seq), multimapping reads are typically filtered out, with only uniquely mapped reads considered for duplicate removal^26^. For specialized analyses targeting repetitive regions, dedicated tools are employed^27^. Furthermore, the performance of alignment-based tools is intrinsically dependent on the choice of the mapping program, its parameters, and the quality of the reference genome assembly, introducing additional variables that complicate direct comparisons. Collectively, these considerations demonstrate that conducting a controlled and equitable comparison of duplicate removal efficacy is particularly challenging, and this issue warrants a separate in-depth investigation (see, for example^12^,).

**Fig. 5:**
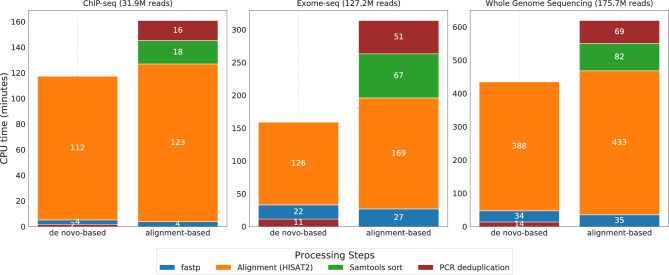
CPU time of pipelines incorporating *de novo*-based versus alignment-based deduplication approaches. PCR duplicates were identified with zero mismatches allowed. Each value corresponds to the median of five runs of the corresponding tool. The order of processing steps from bottom to top reflects the sequential order of program execution in the respective pipelines.

In this work, we compared the CPU time and RAM of NGS data processing pipelines integrating either a *de novo*-based deduplication method (Fastq-dupaway “tight”, fastp^28^, HISAT2^29^) or an alignment-based one (fastp, HISAT2, Samtools sort, Picard MarkDuplicates). The results of testing on datasets of different sizes demonstrate that the alignment-based pipeline requires significantly more CPU time and RAM to complete than the *de novo*-based pipeline (Fig. 5, Fig. S6, Fig. S7, Table S9, Table S10, Table S11). The main contribution to this slowdown came from the stage of sorting mapped reads (Samtools sort), which is mandatory for the alignment-based approach, as well as the relatively low speed of the Picard MarkDuplicates algorithm. Another contributing factor, albeit less significant, was the reduction in mapping time when reads are deduplicated at the FASTQ-file stage (*de novo*-based approach) before alignment, as opposed to mapping the entire original dataset. As expected, the higher the initial proportion of PCR duplicates, the more pronounced this difference becomes (Fig. S8, Table S9, Table S11).

In conclusion, the alignment-based deduplication procedure is computationally expensive and requires significant time, which limits its practical application for the analysis of large NGS data sets under conditions of limited computing resources.

## Discussion

Our comprehensive comparison of *de novo*-based PCR duplicate removal tools reveals a critical computational bottleneck: while these tools achieve high accuracy, their application to large datasets is severely constrained by memory requirements that scale with input size. For instance, processing a 538 GB dataset required tools like CD-HIT-DUP and FastUniq to consume up to 1 TB of RAM, making them impractical for researchers without access to high-performance computing infrastructure. To address this limitation, we developed Fastq-dupaway, a novel tool implementing a new deduplication approach that substantially reduces RAM usage while maintaining accuracy and processing speed. In its “tight”, “loose”, and “tail-hamming” modes, Fastq-dupaway maintains a nearly constant RAM footprint of approximately 2 GB, enabling deduplication of very large datasets even on personal computers.

Runtime comparisons revealed that Fastq-dupaway in “fast” mode offers the fastest deduplication performance, surpassed in memory efficiency only by Seqkit rmdup, which is limited to single-end reads. For paired-end data, common in most modern NGS experiments, FastUniq is a widely used *de novo*-based tool for removing PCR duplicates (more than 500 citations). Our Fastq-dupaway tool in “loose” mode matches FastUniq’s functionality while remaining fast and RAM-efficient, making it the optimal solution among the tested tools. It is important to note that Fastq-dupaway in its “sequence-based” modes creates temporary files totaling approximately 2x the input file size. The computational complexity of our algorithm can be estimated as $O(N\cdot \log N$).

Another important aspect is the influence of algorithmic implementation on performance stability. Fastq-dupaway, with its optimized C++ implementation, exhibits consistently low runtime variability across multiple runs, a critical feature for practical applications where predictable performance is essential.

Furthermore, analysis of duplicate removal with an allowable mismatch level (e.g., two nucleotides) highlighted that different programs exhibit varying “stringency” in deduplication, driven by the transitivity issue in read distance calculations. Our analysis revealed that in the absence of sequencing errors, the results of all programs are consistent; however, allowing for two mismatches reveals substantial differences in their outputs. This finding underscores the need for standardized deduplication algorithms in the scientific community to ensure comparability across studies.

Our findings demonstrate the computational advantage of the *de novo*-based deduplication approach over the alignment-based method. The necessity of the sorting step and the relatively low execution speed of the Picard MarkDuplicates algorithm make the alignment-based pipeline substantially more resource-intensive. The choice between these approaches, however, depends on a multitude of factors, including available computational resources, sequencing coverage, the type and parameters of the aligner, genomic variability (e.g., SNP density), and the availability of a high-quality reference genome. Therefore, for research tasks where the use of alignment-based tools (such as the Picard toolkit) is not strictly required by the experimental design, we recommend employing *de novo*-based tools such as Fastq-dupaway. This choice allows for a drastic reduction in processing time and computational costs, especially when working with large-scale NGS datasets, without compromising the core objective of PCR duplicate removal in standard analyses.

Our study evaluated computational efficiency but could not assess accuracy (false positive/negative rates) due to the absence of ground truth data. The accuracy validation of Fastq-dupaway was performed indirectly by comparing Fastq-dupaway’s results with those of established tools widely used in biomedical research practice. Fastq-dupaway removes a similar number of duplicates as other programs. Overall, our tests demonstrate that Fastq-dupaway is a versatile and resource-efficient tool for removing PCR duplicates from both single-end and paired-end NGS data, particularly for large datasets processed on resource-constrained platforms. This positions it as a promising solution for a wide range of biological and medical research applications.

## Methods

### Benchmarking environment

All tool runs were performed on a high-performance computing server equipped with an Intel$\mathrm{\textregistered}$ Xeon$\mathrm{\textregistered}$ Gold 6226 CPU @ 2.70GHz and 1.5 TB RAM, running on a GPFS cluster. GPFS is backed by enterprise-grade 14 TB HDD SAS 7200 RPM hard drive shelves configured with RAID 6 for performance, connected via InfiniBand to “dss” servers. Sequential I/O tests using command dd on the GPFS filesystem showed write speeds of 1.3 GB/s and read speeds of 1.1 GB/s.

#### Benchmarking datasets

All datasets used in this benchmarking study are publicly available through the NCBI Sequence Read Archive (https://www.ncbi.nlm.nih.gov/sra). Dataset accession numbers and experimental details are provided in Table 2.

#### Launching programs

All PCR deduplication tools were executed on a single CPU core (supporting one thread) to ensure equitable comparison between BBTools clumpify (which supports multi-core processing) and other tools lacking this capability. Each program was run five times on every dataset to ensure measurement reliability and account for potential performance variability.

Performance metrics – including User time, System time, Elapsed time, and memory usage (resident set size, RSS) – were measured using the /usr/bin/time Unix command. CPU time was calculated as the sum of User and System time. Complete program launch parameters and versions are provided in the supplementary materials.

## Supplementary Information

Supplementary Information 1.

Supplementary Information 2.

## Data Availability

The datasets analysed during the current study are publicly available. They can be downloaded from the Sequence Read Archive (SRA) using the identifiers in Table 2. Artificial data was generated by commands presented in supplementary materials.
