# Supplementary material for: Fastq-dupaway: a fast and memory-efficient tool for deduplication of single- and paired-end NGS data
Source: Sci Rep. 2025 Nov 25;15:45303. doi: 10.1038/s41598-025-28948-w (PMC12749013; doi:10.1038/s41598-025-28948-w)
Supplement: Supplementary file 1 — Supplementary Information 1. [file 41598_2025_28948_MOESM1_ESM.pdf]

Supplementary Materials: Fastq-dupaway – a fast and memory-efficient tool for efficient deduplication of single- and paired-end NGS data

Launching programs

Comparison with *de novo*-based PCR deduplication tools. Exact commands used in experiment

- Fastq-dupaway (v. 1.3)

  For paired-end reads data:

  ```
  ./fastq-dupaway -i SRR19505554_1.fastq -u SRR19505554_2.fastq -o <output1> -p <output2> # "tight" mode
  ```

  ```
  ./fastq-dupaway -i SRR19505554_1.fastq -u SRR19505554_2.fastq -o <output1> -p <output2> -compare-seq "loose"
  ```

  ```
  ./fastq-dupaway -i SRR19505554_1.fastq -u SRR19505554_2.fastq -o <output1> -p <output2> -compare-seq "tail-hamming" -distance <mismatch> # number of mismatches is either 0 or 2
  ```

  ```
  ./fastq-dupaway -i SRR19505554_1.fastq -u SRR19505554_2.fastq -o <output1> -p <output2> -fast
  ```

  For single-end reads data:

  ```
  ./fastq-dupaway -i SRR10044362.fastq -o <output> # "tight" mode
  ```

  ```
  ./fastq-dupaway -i SRR10044362.fastq -o <output> -compare-seq "loose"
  ```

  ```
  ./fastq-dupaway -i SRR10044362.fastq -o <output> -compare-seq "tail-hamming" -distance <mismatch> # number of mismatches is either 0 or 2
  ```

  ```
  ./fastq-dupaway -i SRR10044362.fastq -o <output> -fast
  ```

- CD-HIT-DUP (v. 4.8.1)

  For paired-end reads data (number of mismatches is either 0 or 2):

  ```
  ./cd-hit-dup -i SRR19505554_1.fastq -i2 SRR19505554_2.fastq -o <output1> -o2 <output2> -e <mismatch>
  ```

  For single-end reads data (number of mismatches is either 0 or 2):

  ```
  ./cd-hit-dup -i SRR10044362.fastq -o <output> -e <mismatch>
  ```

- FastUniq (v. 1.1)

  For paired-end reads data:

  ```
  ./fastuniq -i input_srr_list.txt -o <output1> -p <output2>
  ```

- BBtools Clumpify (v. 39.06)

  For paired-end reads data (number of mismatches is either 0 or 2):

  ```
  ./clumpify.sh in=SRR19505554_1.fastq in2=SRR19505554_2.fastq dedupe=t subs=<mismatch> out=<output1> out2=<output2> t=1
  ```

  For single-end reads data (number of mismatches is either 0 or 2):

  ```
  ./clumpify.sh in=SRR10044362.fastq dedupe=t subs=<mismatch> out=<output> t=1
  ```

- Fastx Toolkit Collapser (v. 0.0.14)

  For single-end reads data:

  ```
  ./fastx_collapser -v -i SRR10044362.fastq -o <output>
  ```

- Seqkit rmdup (v. 2.8.2)

  For single-end reads data:

  ```
  ./seqkit rmdup -s -o <output> < SRR10044362.fastq
  ```

Comparison with the alignment-based PCR deduplication tool. Exact commands used in experiment

- Fastp (v. 0.23.2)

```
./fastp -i SRR19505554_1.fastq -I SRR19505554_1.fastq -o SRR19505554_1.trimmed.fastq
-O SRR19505554_2.trimmed.fastq -w 1
```

- Hisat2 (v. 2.2.1, human genome version - GRCh38.p13 (hg38), mouse genome version - GRCm38.p6 (mm10))

```
./hisat2 -p 1  --rg-id MY\_GROUP --rg \textbackslash SM:sample\_name\textbackslash
-x \$reference\_path -1 SRR19505554\_1.trimmed.fastq
-2 SRR19505554\_2.trimmed.fastq | samtools view -bS -o SRR19505554\_trimmed.bam
```

- Samtools sort (v. 1.22)

```
./samtools sort -o SRR19505554_sorted.bam SRR19505554_trimmed.bam
```

- Picard MarkDuplicates (v. 3.4.0)

```
java -jar picard.jar MarkDuplicates I=SRR19505554_sorted.bam O=SRR19505554_markdup.bam
```

- Fastq-dupaway (v. 1.3)

```
./fastq-dupaway -i SRR19505554_1.fastq -u SRR19505554_2.fastq -o <output1> -p <output2>
```

Python and Bash code to generate datasets with 6.8 and 12.7% PCR duplicates based on the SRR13232316 dataset for Figure S9

- Running docker fastq-dupaway ”tight”:

```
    export WORKDIR=/home/sra/SRR13232316

    docker run -it --rm -v ${WORKDIR}:/data fastq-dupaway -i
    /data/SRR13232316_1.fastq -u /data/SRR13232316_2.fastq -o
    /data/deduplicated_SRR13232316_1.fastq -p
    /data/deduplicated_SRR13232316_2.fastq --compare-seq tight
```

- Bash:

```
    grep "^@SRR" deduplicated_SRR13232316_1.fastq >
    srr_ids_deduplicated_SRR13232316_1.txt

    grep "^@SRR" SRR13232316_1.fastq > srr_ids_SRR13232316_1.txt
```

- Python:

```
dir_path = "/home/sra/SRR13232316"

def load_ids(filename):
    with open(filename) as f:
        return {line.split()[0] for line in f if line.startswith('@')}

original_ids = load_ids(f'{dir_path}/srr_ids_SRR13232316_1.txt')

deduplicated_ids = load_ids(f'{dir_path}/srr_ids_deduplicated_SRR13232316_1.txt')

pcr_duplicate_ids = original_ids - deduplicated_ids

six_percent_PCR_dublicates = list(pcr_duplicate_ids)[:7631871]

all_srrIDS_with_six_percent_PCR_dublicates = six_percent_PCR_dublicates +
```

```
list(deduplicated_ids)

all_srrIDS_with_12_percent_PCR_dublicates = all_srrIDS_with_six_percent_PCR_dublicates +
list(set(pcr_duplicate_ids) - set(six_percent_PCR_dublicates))[:7631871]

file1 = open(f'{dir_path}/ids_for_seqkit_6percent_PCR_dublicates.txt', 'w')
for i in all_srrIDS_with_six_percent_PCR_dublicates:
    file1.write(i.split("@")[1] + "\n")
file1.close()

file1 = open(f'{dir_path}/ids_for_seqkit_12percent_PCR_dublicates.txt', 'w')
for i in all_srrIDS_with_12_percent_PCR_dublicates:
    file1.write(i.split("@")[1] + "\n")
file1.close()
```

- Bash:

```
seqkit grep -j 10 -f ids_for_seqkit_6percent_PCR_dublicates.txt
SRR13232316_1.fastq -o SRR13232316_1_with_six_percent_PCR_dublicates.fastq

seqkit grep -j 10 -f ids_for_seqkit_6percent_PCR_dublicates.txt
SRR13232316_2.fastq -o SRR13232316_2_with_six_percent_PCR_dublicates.fastq

seqkit grep -j 10 -f ids_for_seqkit_12percent_PCR_dublicates.txt
SRR13232316_1.fastq -o SRR13232316_1_with_12percent_PCR_dublicates.fastq

seqkit grep -j 10 -f ids_for_seqkit_12percent_PCR_dublicates.txt
SRR13232316_2.fastq -o SRR13232316_2_with_12percent_PCR_dublicates.fastq
```

Supplementary Note 1

The analysis of triple-metric performance comparison of deduplication tools using mixed linear models. Performance comparisons among deduplication tools were conducted using mixed linear models across three metrics: Elapsed Time, CPU Time, and Peak Memory Consumption. Pairwise differences between fastq-dupaway in "fast" mode and all other tools were assessed by

- Fitting a mixed linear model with tool as a fixed effect and dataset as a random effect to estimate mean performance per tool.
- Computing Estimated Marginal Means for pairwise comparisons.

All analyses were performed in Python using the statsmodels library (version 0.14.5). Input performance data were log-transformed, and diagnostic tests confirmed that model residuals satisfied assumptions of normality and homoscedasticity. Results showing mean difference, 95% CI and adjusted p-value for every pairwise comparison of tools with Fastq-dupaway "fast" are presented in tables 1, 2, 3.

Contrast	MeanDiff	95% CI Low	95% CI High	p-value	p_adj(Bonferonni)
Fastq-dupaway "fast" vs BBTools Clumpify	1.90	1.26	2.54	5.3e-09	1.91e-07
Fastq-dupaway "fast" vs CD-HIT-DUP	1.54	0.90	2.17	2.4e-06	8.5e-05
Fastq-dupaway "fast" vs Fastq-dupaway "loose"	1.81	1.17	2.45	2.8e-08	1.0e-06
Fastq-dupaway "fast" vs Fastq-dupaway"tail-hamming"	1.80	1.15	2.43	3.8e-08	1.36e-06
Fastq-dupaway "fast" vs Fastq-dupaway"tigh"	1.73	1.09	2.37	1.0e-07	3.6e-06
Fastq-dupaway "fast" vs Fastuniq	2.19	1.56	2.84	1.7e-11	6.3e-10
Fastq-dupaway "fast" vs Fastx Toolkit Collapser	1.04	0.37	1.70	2.4e-03	8.5e-02
Fastq-dupaway "fast" vs Seqkit rmdup	2.08	1.41	2.75	9.8e-10	3.5e-08

Table 1. Pairwise comparisons between Fastq-dupaway "fast" and all other tested tools on the Elapsed Time data.

Contrast	MeanDiff	95% CI Low	95% CI High	p-value	p_adj(Bonferonni)
Fastq-dupaway "fast" vs BBTools Clumpify	0.93	0.33	1.54	2.5e-03	9.0e-02
Fastq-dupaway "fast" vs CD-HIT-DUP	0.64	0.03	1.25	3.8e-02	1.0e+00
Fastq-dupaway "fast" vs Fastq-dupaway "loose"	1.89	1.28	2.49	1.0e-09	3.6e-08
Fastq-dupaway "fast" vs Fastq-dupaway"tail-hamming"	1.86	1.25	2.47	1.8e-09	6.5e-08
Fastq-dupaway "fast" vs Fastq-dupaway"tigh"	1.88	1.27	2.49	1.2e-09	4.2e-08
Fastq-dupaway "fast" vs Fastuniq	1.22	0.61	1.82	8.4e-05	3.0e-03
Fastq-dupaway "fast" vs Fastx Toolkit Collapser	0.21	-0.41	0.82	5.1e-01	1.0e+00
Fastq-dupaway "fast" vs Seqkit rmdup	1.49	0.87	2.10	2.4e-06	8.8e-05

Table 2. Pairwise comparisons between Fastq-dupaway "fast" and all other tested tools on the CPU Time data.

Contrast	MeanDiff	95% CI Low	95% CI High	p-value	p_adj(Bonferonni)
Fastq-dupaway "fast" vs BBTools Clumpify	1.43	0.72	2.14	7.7e-05	2.8e-03
Fastq-dupaway "fast" vs CD-HIT-DUP	1.24	0.53	1.93	5.6e-04	2.0e-02
Fastq-dupaway "fast" vs Fastq-dupaway "loose"	5.27	4.57	5.98	0.0e+00	0.0e+00
Fastq-dupaway "fast" vs Fastq-dupaway"tail-hamming"	5.27	4.57	5.98	0.0e+00	0.0e+00
Fastq-dupaway "fast" vs Fastq-dupaway"tigh"	5.27	4.57	5.98	0.0e+00	0.0e+00
Fastq-dupaway "fast" vs Fastuniq	1.57	0.85	2.29	2.0e-05	7.3e-04
Fastq-dupaway "fast" vs Fastx Toolkit Collapser	3.03	2.09	3.97	2.8e-10	1.0
Fastq-dupaway "fast" vs Seqkit rmdup	4.28	3.34	5.22	0.0e+00	0.0e+00

Table 3. Pairwise comparisons between Fastq-dupaway "fast" and all other tested tools on the Peak Memory Consumption data.

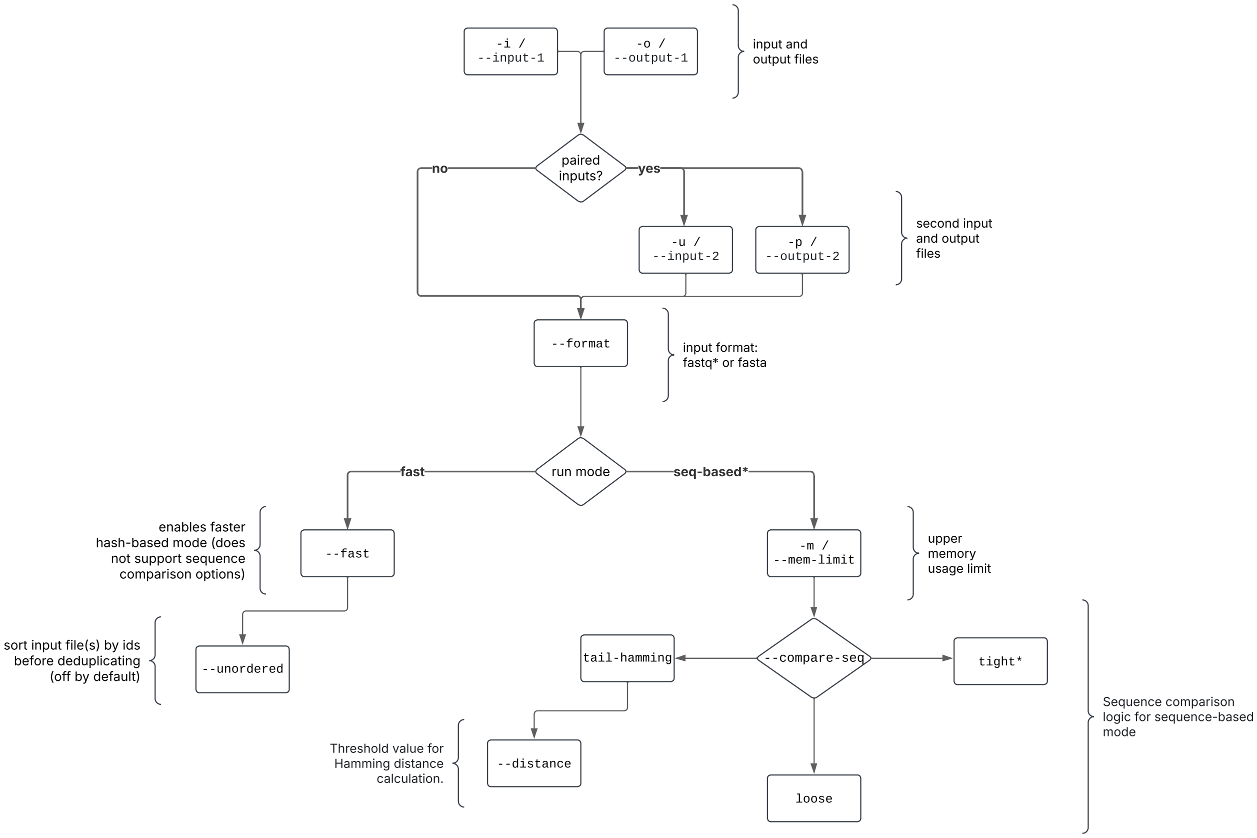

Figure S1. Block diagram with all available options of the Fastq-dupaway program. Options enabled by default are marked with “*”.

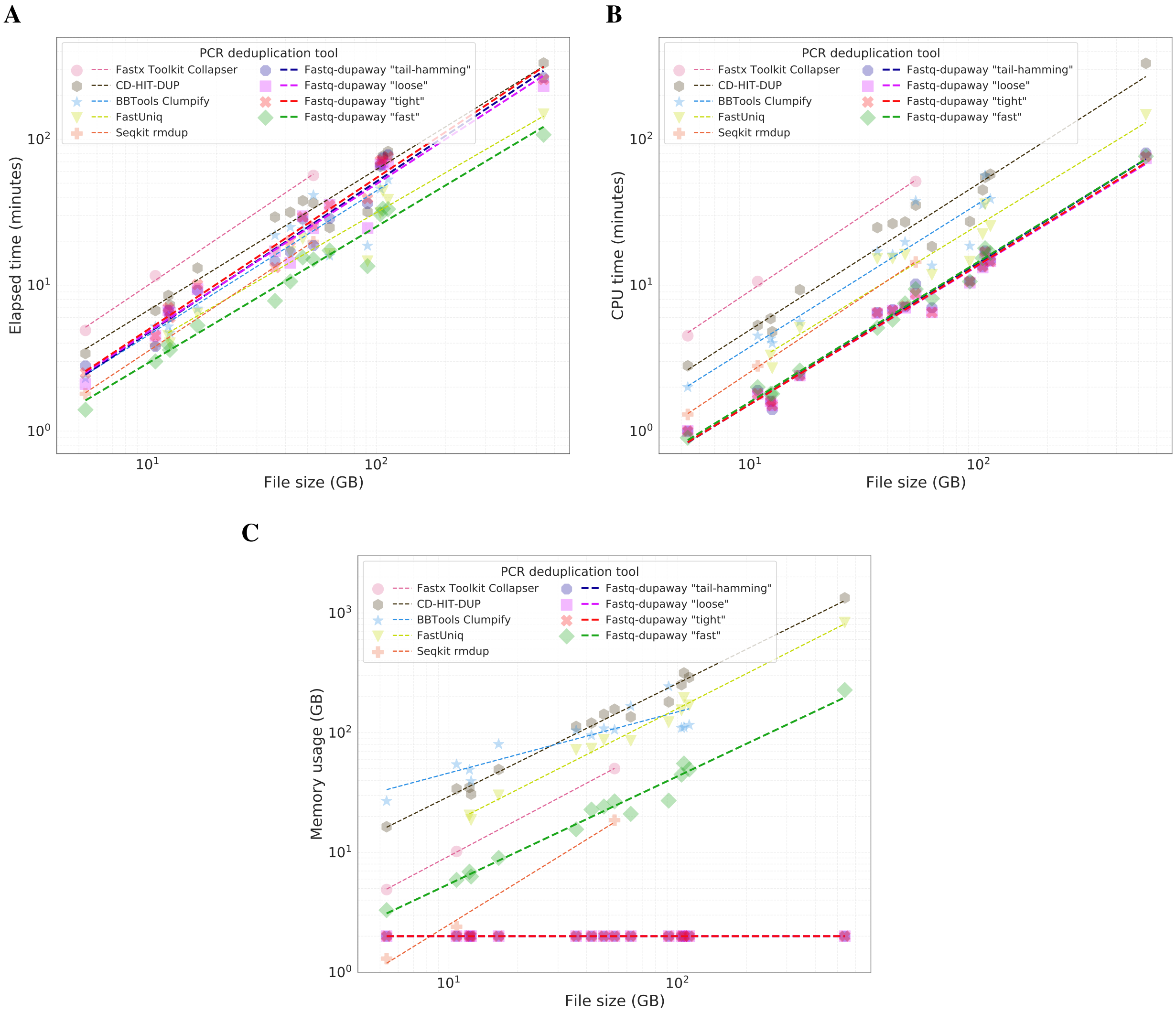

Figure S2. Evaluation of (A) Elapsed time, (B) CPU time and (C) memory usage for each PCR deduplication tool when processing datasets of different sizes. PCR duplicates were identified with zero mismatches allowed. Each point corresponds to the median of five runs of the corresponding tool. BBTools Clumpify does not have a point for the "Hi-C (538 GB)" data because the program terminated with an error.

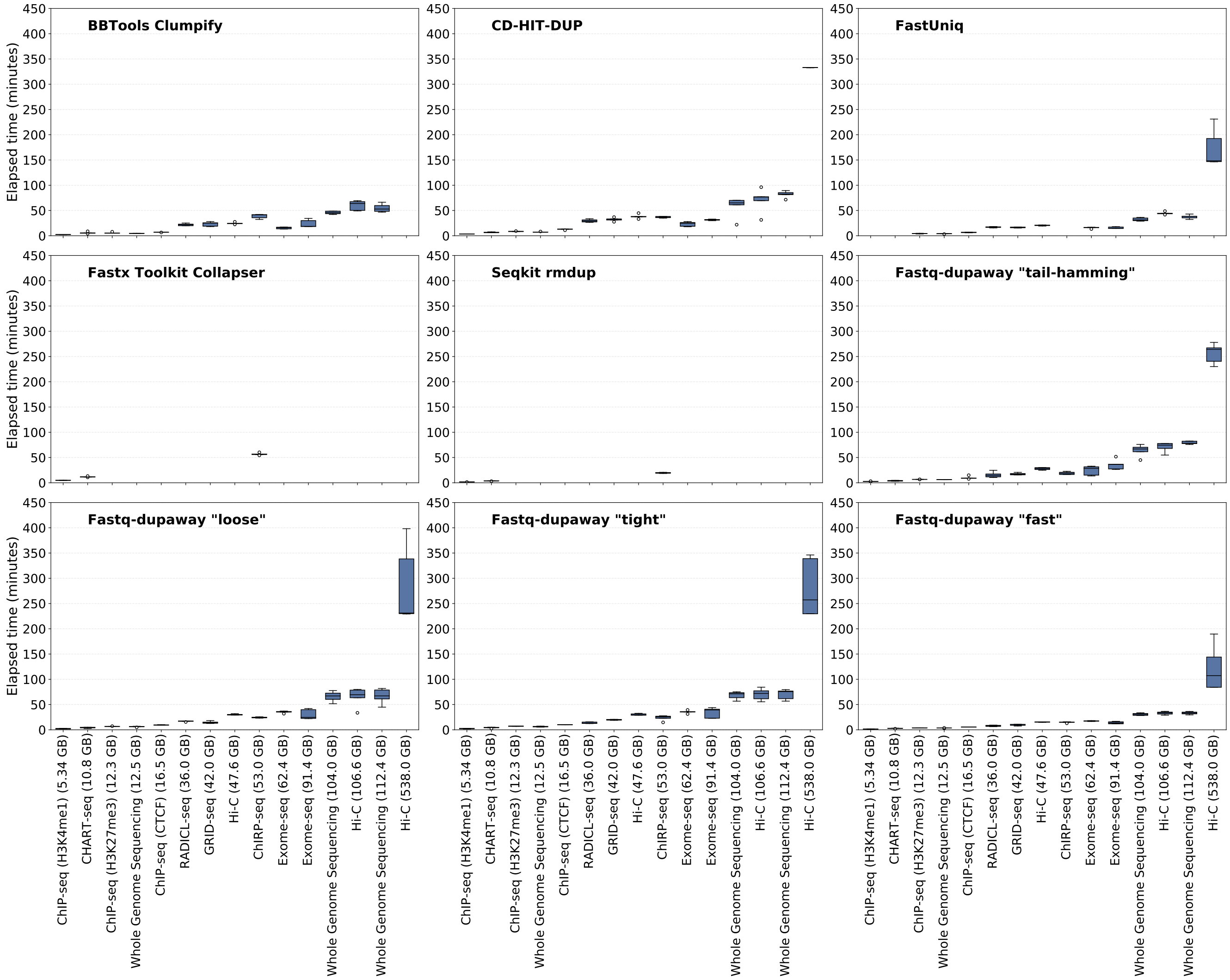

Figure S3. Distribution of elapsed time of tools launched 5 times on each dataset. PCR duplicates were identified with zero mismatches allowed. BBTools Clumpify does not have a boxplot for the “Hi-C (538 GB)” data because the program terminated with an error. FastUniq, Fastx Toolkit Collapser, Seqkit rmdup do not produce boxplots for datasets not supported by those tools.

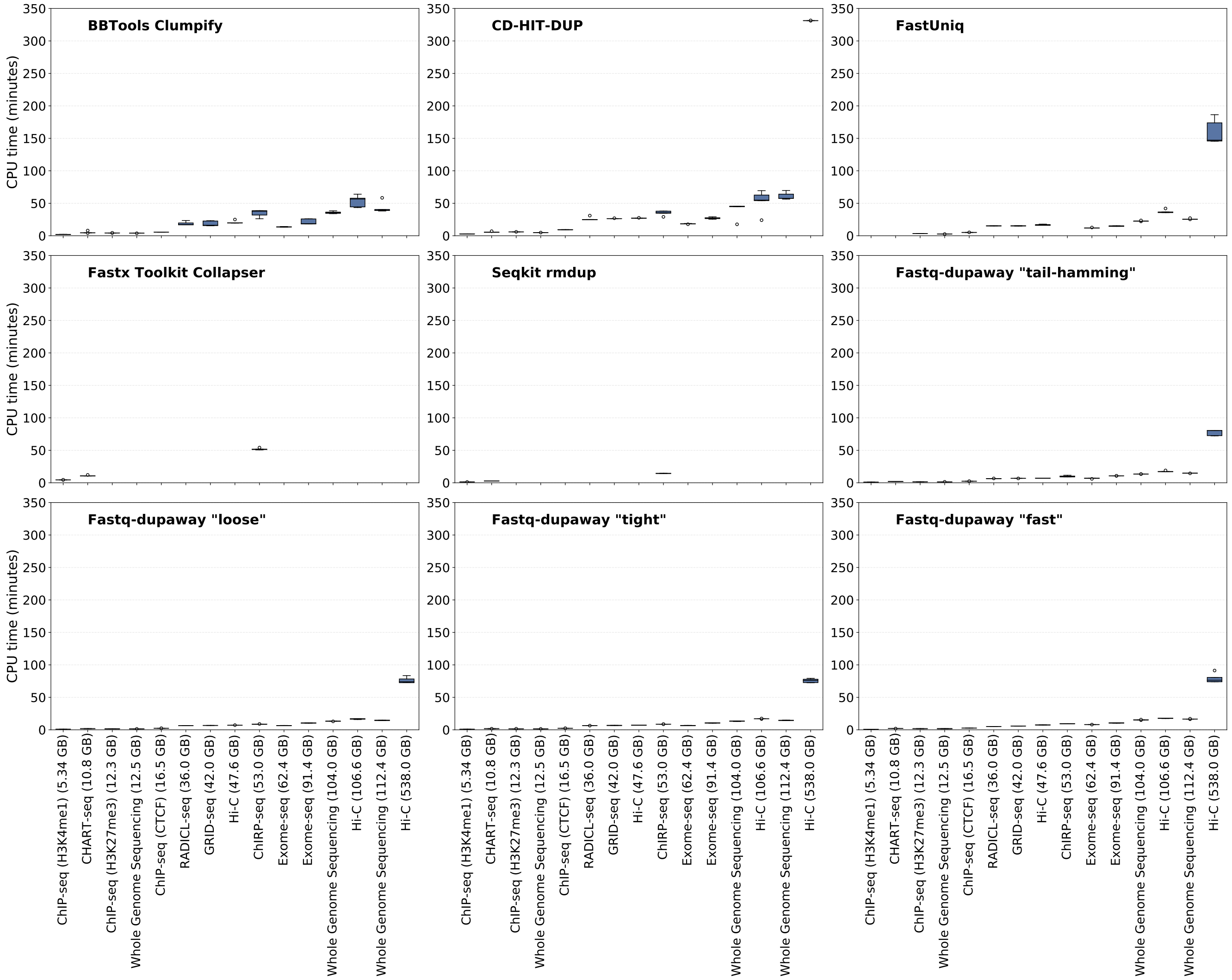

Figure S4. Distribution of CPU time of tools launched 5 times on each dataset. PCR duplicates were identified with zero mismatches allowed. BBTools Clumpify does not have a boxplot for the “Hi-C (538 GB)” data because the program terminated with an error. FastUniq, Fastx Toolkit Collapser, Seqkit rmdup do not produce boxplots for datasets not supported by those tools.

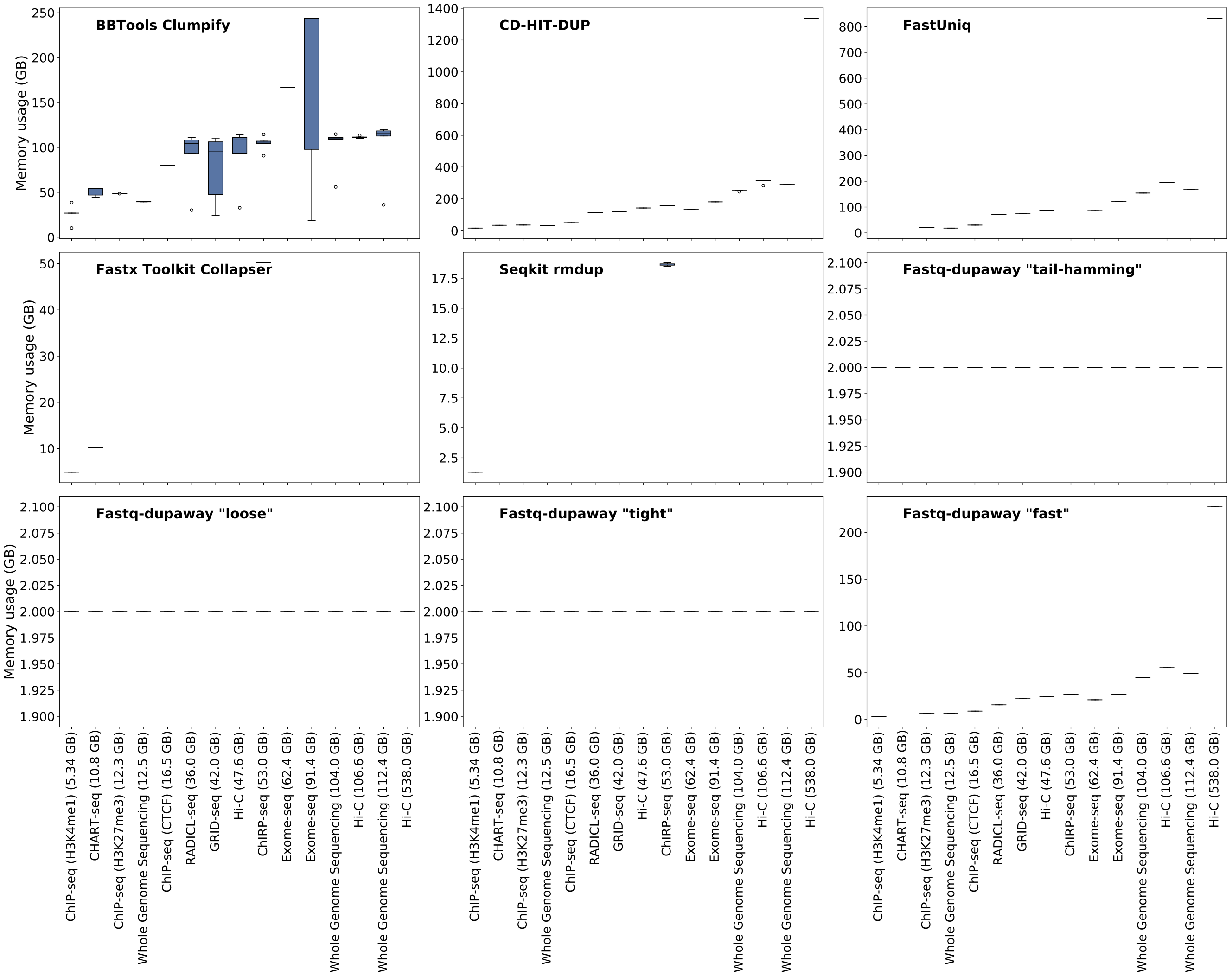

Figure S5. Distribution of memory usage of tools launched 5 times on each dataset. PCR duplicates were identified with zero mismatches allowed. BBTools Clumpify does not have a boxplot for the "Hi-C (538 GB)" data because the program terminated with an error. FastUniq, Fastx Toolkit Collapser, Seqkit rmdup do not produce boxplots for datasets not supported by those tools.

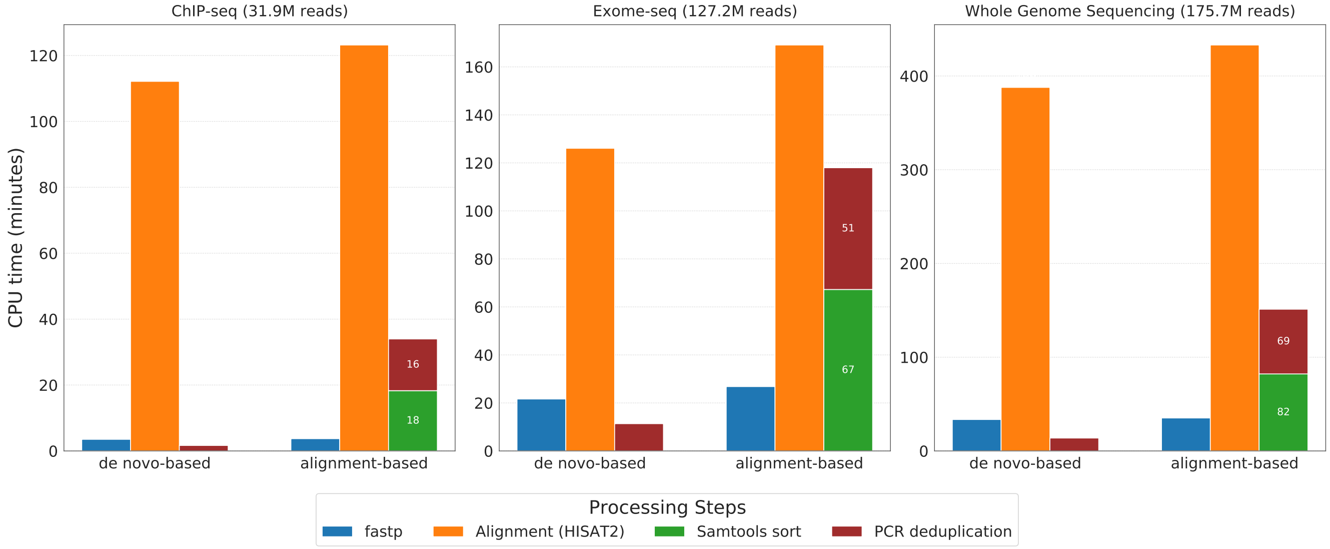

Figure S6. CPU time of pipelines incorporating *de novo*-based versus alignment-based deduplication approaches. PCR duplicates were identified with zero mismatches allowed. Each value corresponds to the median of five runs of the corresponding tool.

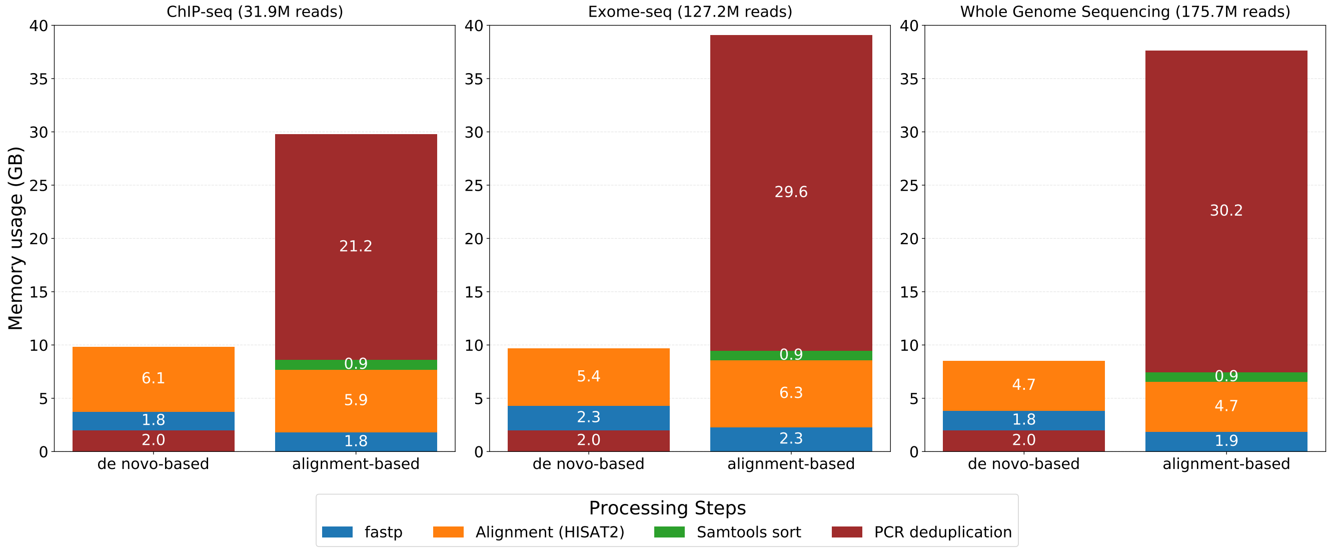

Figure S7. Memory usage (GB) of pipelines incorporating *de novo*-based versus alignment-based deduplication approaches. PCR duplicates were identified with zero mismatches allowed. Each value corresponds to the median of five runs of the corresponding tool. The order of processing steps from bottom to top reflects the sequential order of program execution in the respective pipelines.

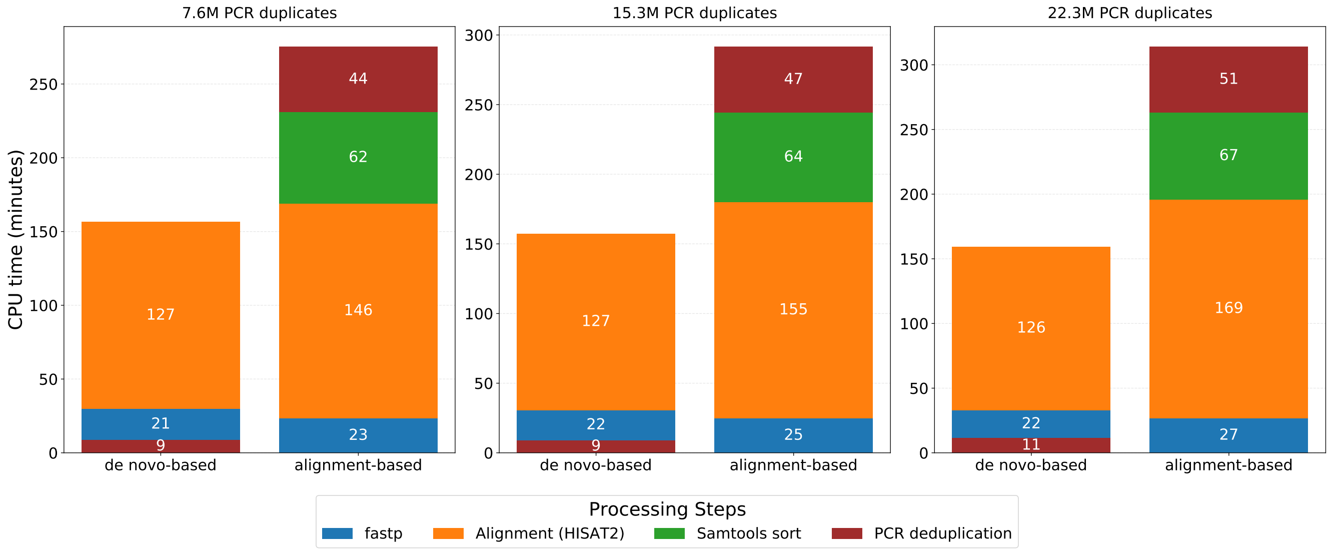

Figure S8. CPU time of pipelines incorporating *de novo*-based versus alignment-based deduplication approaches. PCR duplicates were identified with zero mismatches allowed. Data are presented for three datasets with different numbers of PCR duplicates obtained from SRR13232316: (left) ∼6.8% duplicates, (center) ∼12.7% duplicates, (right) original SRR13232316 library with ∼17.5% duplicates. Across all these datasets, 104.9 million reads are common and are not identified as PCR duplicates by Fastq-dupaway "tight" standards.
